# Surgical and Neurological Outcomes in Robotic Thymectomy for Myasthenic Patients with Thymoma

**DOI:** 10.3390/life15030371

**Published:** 2025-02-26

**Authors:** Khrystyna Kuzmych, Dania Nachira, Amelia Evoli, Raffaele Iorio, Carolina Sassorossi, Maria Teresa Congedo, Gregorio Spagni, Alessia Senatore, Giuseppe Calabrese, Stefano Margaritora, Elisa Meacci

**Affiliations:** 1 Department of General Thoracic Surgery, Fondazione Policlinico Universitario “A. Gemelli”, IRCCS, Università Cattolica del Sacro Cuore, 00168 Rome, Italy; dania.nachira@policlinicogemelli.it (D.N.); sassorossi.caro@gmail.com (C.S.); mariateresa.congedo@policlinicogemelli.it (M.T.C.); alessiasenatore2@gmail.com (A.S.); giuseppe.calabrese@guest.policlinicogemelli.it (G.C.); stefano.margaritora@unicatt.it (S.M.); elisa.meacci@unicatt.it (E.M.); 2 Institute of Neurology, Fondazione Policlinico Universitario “A. Gemelli”, IRCCS, Università Cattolica del Sacro Cuore, 00168 Rome, Italy; amelia.evoli@unicatt.it (A.E.); raffaele.iorio@policlinicogemelli.it (R.I.); gregorio.spagni@unifi.it (G.S.)

**Keywords:** myasthenia gravis, thymoma, thymectomy, robotic thymectomy, robotic thoracic surgery

## Abstract

Background: While the safety and feasibility of robotic thymectomy have been well documented through several studies, the surgical and long-term neurological outcomes in patients with thymomatous myasthenia gravis (MG), particularly in advanced stages, remain scarce. This study aims to evaluate the surgical outcomes in patients undergoing robotic-assisted thymectomy (RATS) for thymoma and to analyze neurological outcomes in patients with myasthenia. Material and Methods: Out of 128 robotic thymectomies performed at our institution between October 2013 and January 2022, clinical and pathological data from 55 patients diagnosed with thymoma were reviewed. Of these, thirty (54.5%) patients had concomitant acetylcholine-receptor-antibody-associated MG. Neurological outcomes were assessed using the Myasthenia Gravis Foundation of America post-intervention score (MGFA-PIS). Results: Thirty-nine (70.9%) procedures were performed using the left-sided approach. The mean operative time was 196.9 ± 79.9 min in patients with MG compared to 175.8 ± 61.6 min in non-MG patients (*p* = 0.285). Additionally, patients with MG had a longer in-hospital stay (4.8 ± 2.6 vs. 3.3 ± 2.2 days, *p* = 0.01) and a significantly higher need for intensive care unit admission (*p* < 0.01). No deaths were reported. The rates of conversions (3.3% vs. 4.0%, *p* = 0.895) and complications (*p* = 0.813) were comparable between the myasthenic and non-myasthenic thymomas. A multivariable analysis identified lung involvement (*p* = 0.023), vascular involvement (*p* = 0.04), and extended resection (*p* = 0.019) as significant risk factors for conversion and complications. The mean age of surgery for patients with MG was 54.5 ± 15.9 years. After a mean follow-up period of 35.6 ± 25.7 months, 18 (60%) patients with myasthenia showed clinical improvement of their condition. Specifically, 2 patients (6.6%) achieved complete stable remission (CSR), 2 (6.6%) experienced pharmacological remission (PR), 12 (40.0%) demonstrated minimal manifestation (MM), and 4 (13.3%) exhibited a combination of PR and MM. Twelve patients (40%) exhibited no changes, maintaining a stable clinical condition. No clinical worsening was observed. The overall improvement rates at 2 years and 5 years were 38% and 83%, respectively. Conclusions: RATS thymectomy is a safe and feasible approach for patients with thymoma. Patients with coexisting MG may benefit through a good rate of neurological improvement.

## 1. Introduction

Thymoma is a rare epithelial tumour of the thymus gland, typically characterized by slow growth, local invasiveness, and rare distant metastases. It is frequently associated with various types of disorders, most notably myasthenia gravis (MG), which is present in about 20–25% of patients with thymomas. Conversely, thymomas are present in 10–20% of patients with myasthenia [1]. Although thymomas are considered low-grade malignant tumours for which surgical resection is the standard of care, the coexistence of MG complicates their management. Previous studies have shown that MG serves as a marker of poor prognosis in patients with thymoma [2,3].

“Extended thymectomy” is considered the gold standard for thymoma treatment [4,5] and plays a crucial role in the multidisciplinary management of MG [6].

The surgical procedure involves a complete en bloc excision of the tumour, thymus, and perithymic adipose tissue. According to the ITMIG guidelines, the complete removal of anterior mediastinal fat tissue is essential following the identification of the right and left phrenic nerves, extending the dissection from the jugular notch to the anterior pericardiophrenic angle, ensuring both oncological and neurological radicality [7].

Historically, sternotomy has long been the standard approach for thymic epithelial tumours (TETs) and remains the preferred choice in complex cases [8] due to its effectiveness in ensuring radical dissection of the thymus and perithymic fat tissue. However, ongoing debates regarding the best surgical approach have emerged, particularly in the context of minimally invasive techniques. In recent years, given the younger age profile of patients with MG, there has been increasing interest in adopting less invasive methods or thymoma treatment.

Following early evidence of significant advantages from transcervical and video-assisted thoracoscopic surgery (VATS) thymectomy, many surgeons remain hesitant to adopt a minimally invasive technique in myasthenic patients largely due to concerns about the adequacy of tissue resection [9]. Each surgical approach offers its own benefits and drawbacks. The present literature mainly comprises nonrandomized retrospective case series comparing operative approaches. However, the heterogeneity of data, evaluation techniques, and a multitude of confounding factors have rendered direct comparisons difficult if not entirely unfeasible [10]. All of these factors are responsible for the difficulties that arise in achieving a consensus in treatment protocols.

Minimally invasive approaches have consistently proven their effectiveness for patients, particularly those with early-stage disease or for cases with minimal extension to nearby structures, demonstrating oncological outcomes on par with traditional methods. Moreover, they often provide better surgical results, particularly regarding postoperative recovery, with reduced complication rates and shorter hospital stays [11,12,13,14,15,16].

Since then, the introduction of robotic surgery has shown several advantages. The three-dimensional vision system, including articulated instruments with 360 degrees of rotation, seven degrees of freedom, and the tremor filtering system of the da Vinci Surgical Robotic System (Intuitive Surgical, Inc., Sunnyvale, CA, USA), allow for an intuitive intervention with an “open-like” view. The use of CO_2_ insufflation further enhances surgical comfort and visualization in a narrow space, like the mediastinum [17].

Robotic thymectomy has thus emerged as a valid alternative to open approaches [17,18]. Since the first description of this technique for neurological MG treatment, the robotic technique has been extended to thymic epithelial tumours, including not only in early-stage tumours but also those with limited infiltration of surrounding structures [11].

While the safety and feasibility of robotic thymectomy have been well documented through several experiences reported in the literature, reports on long-term neurological outcomes in patients with thymoma with MG, particularly in advanced stages, remain scarce [14,19,20,21].

The aims of this study are to investigate the surgical outcomes of all patients who underwent RATS for thymoma and to evaluate the long-term neurological outcomes in patients with concomitant MG.

## 2. Materials and Methods

This research was structured as a retrospective, single-centre observational study. The study was approved by the Ethical Committee (Università Cattolica del Sacro Cuore, Approval Code: 2020/8903; Approval Date: 19 January 2020) and was therefore conducted in full accordance with the ethical standards outlined in the Declaration of Helsinki and its later amendments.

### 2.1. Study Population

From October 2013 to January 2022, clinical data from 128 patients who underwent thymectomy at our centre were reviewed. For this study, only patients with confirmed pathological diagnosis of thymoma were included. The data and results were reported in accordance with the Strengthening the Reporting of Observational Studies (STROBE) checklist.

Preoperative radiologic assessments were performed using at least one computed tomography (CT) scan or a magnetic resonance imaging (MRI) scan of the thorax.

The inclusion criteria of the study were as follows:

- Thymectomy for pathologically confirmed thymoma;
- Myasthenic or non-myasthenic patients;
- For MG patients, only those who tested positive for anti-acetylcholine receptor (AChR) antibodies;
- Procedure performed via RATS approach;
- Adult patients (age ≥ 18 years);
- Signed informed consent.

The exclusion criteria included patients affected by thymic carcinoma or benign thymic disease, patients deemed unresectable during surgery, patients lost at follow-up (FUP), and patients with anti-MuSK, anti-titin, or other rare autoantibodies. Additionally, seronegative MG patients were not considered in this analysis.

### 2.2. Neurological Assessment

For patients with MG, the diagnosis was confirmed by neurologists based on standard clinical and laboratory criteria. All patients underwent comprehensive neurological evaluation, including assessment of fluctuating muscle weakness and serological testing for AChR antibodies, as well as electrophysiological testing (EMG) for further confirmation of the clinical diagnosis. The clinical severity of the disease was assessed using the classification system established by the Myasthenia Gravis Foundation of America (MGFA). To assess the impact of disease duration on surgical and neurological outcomes, MG onset until surgery was categorized into the following groups: 0–12 months, 13–24 months, 25–36 months, 37–48 months, and below or equal to 60 months. Also, preoperative MG treatment regimens were recorded, including acetylcholinesterase inhibitors, corticosteroids, immunosuppressive therapy, and intravenous immunoglobulin or plasmapheresis.

Preoperative plasmapheresis was considered for patients with MG who experienced severe symptoms, such as dyspnea, dysphagia, and generalized asthenia, when first-line treatments were insufficient to achieve adequate symptom control or in cases of clinical deterioration. This treatment was implemented as part of preoperative stabilization strategy to optimize patients before thymectomy.

### 2.3. Surgical Technique

The robotic procedures were performed by a single surgeon trained in robotic surgery. Procedure time was defined as the interval from the first incision to the closure of the skin. All surgeries were performed under general anesthesia with single-lung ventilation. A left-side RATS approach was routinely performed, except in cases where preoperative radiological evaluation showed masses entirely projecting onto the right edge of the mediastinum. Three robotic arms were used: the camera port was positioned in the fourth intercostal space along the midaxillary line, and the other two trocars were placed in the third intercostal space along the anterior axillary line and the fifth intercostal space between the midclavicular and the anterior axillary lines [22]. A low-pressure capnothorax was usually induced with 7–8 mmHg of CO_2_. In all analyzed cases, an extended thymectomy was performed following the recommendations of the International Thymic Malignancy Interest Group. The procedure involved identifying the phrenic nerve bilaterally and the innominate vein in the rostral position, followed by extended thymectomy en bloc with mediastinal adipose tissue. Additionally, a thorough exploration of the perithymic lymph node stations was performed as recommended by the nodal mapping protocol of the International Association for the Study of Lung Cancer/International Thymic Malignancy Interest Group [7].

The surgical specimen was removed through an Endo Bag (Medtronic^®^, Minneapolis, MN, USA), with varying sizes and strengths selected based on tumour size. If necessary, the lowest incision was slightly enlarged to facilitate specimen removal. A chest tube was inserted through this same incision. In accordance with the guidelines of the International Thymic Malignancy Interest Group, thymomas were resected using the no-touch and en bloc strategies to minimize tumour manipulation and ensure oncological integrity [23,24].

### 2.4. Postoperative Management and Follow-Up

In the absence of surgical (classified according to the Clavien–Dindo classification) [25] and neurological complications, the patient was discharged after chest tube removal. Operative mortality was defined as death within 30 days after surgery or during the same period of hospitalization.

At histological evaluation of the specimen, thymomas were categorized using the World Health Organization histological classification of thymomas. Tumour invasion was classified by the Masaoka–Koga staging system and TNM classification of malignant tumours [26]. Complete resection (R0) was described as no evidence of residual tumour tissue on surgical margins. Incomplete resection was classified as either microscopic (R1) or macroscopic (R2) evidence of residual tumour tissue.

Patients underwent a clinical–radiological FUP through periodic radiological examinations (such as total-body CT scan) and neurological evaluations.

Overall survival (OS) was defined as the time elapsed between surgery and the date of last follow-up or death, while recurrence-free survival (RFS) was defined as the time between surgery and the first recurrence of disease.

The Myasthenia Gravis Foundation of America post-intervention score (MGFA-pis) was used to assess neurological outcomes [27]. Postoperative radiotherapy was advised based on predefined oncological criteria in accordance with the European Society for Medical Oncology (ESMO) and the National Comprehensive Cancer Network (NCCN) guidelines. The indications for radiotherapy included Masaoka–Koga stages III-IV, WHO B3 histology, and incomplete resection (R1/R2 margins), where micro- or macroscopic residual tumour was present [27].

However, the decision to administer RT in each specific case was made through multidisciplinary team (MDT) discussions, involving thoracic surgeons, oncologists, neurologists, and radiation oncologists.

### 2.5. Statistical Analysis

Continuous variables were expressed as the mean and standard deviation (SD), whereas categorical variables were reported as numbers and percentages. Comparison of categorical variables was performed using the Chi-square test, whereas continuous variables were analyzed using Student’s independent-sample *t*-test or the Mann–Whitney U-test, if normally or non-normally distributed (according to the Shapiro–Wilk test).

Myasthenic improvement, OS and RFS were assessed using Kaplan–Meier curves. A univariable Cox regression was initially performed, and variables exhibiting a *p*-value below 0.20 were included in a subsequent multivariable Cox proportional hazards model to identify independent prognostic factors for conversion and complications. Statistical significance was set at a *p*-value of less than 0.05.

Statistical analysis was performed using the IBM SPSS Statistics for Macintosh, Version 25.00 (Armonk, NY, USA).

## 3. Results

Between October 2013 and January 2022, 128 patients underwent robotic thymectomy at our centre, and 55 of those patients were diagnosed with thymoma. Of this subgroup, 30 patients (54.5%) presented with acetylcholine-receptor-antibody-associated MG. The timing of MG onset prior to surgery varied, with 21 patients (70%) diagnosed within 12 months, 5 (16.7%) between 13 and 24 months, and 5 (13.3%) beyond 24 months, including 2 cases over 5 years. Among these, a detailed breakdown of MGFA classification was performed based on the preoperative disease severity: 6 patients (20%) had Class I (ocular MG), while the remaining 24 (80%) had generalized MG: Class IIa (10%), Class IIb (43.3%), Class IIIa (10%), Class IIIb (10%), and Class IVb (6.7%).

Regarding preoperative medical therapy, 5 patients (16.7%) were managed with anti-AChR monotherapy (pyridostigmine only), while 18 patients (60.0%) received a combination of pyridostigmine and an immunosuppressant. Additionally, seven patients (23.3%) were placed on a triple therapy regimen, including pyridostigmine, corticosteroids (oral only), and azathioprine.

Additionally, four patients (13.3%) underwent preoperative plasmapheresis due to severe symptoms, including dyspnea, dysphagia, and generalized asthenia. Each of these patients underwent five cycles of plasmapheresis, leading to symptom stabilization before surgery with varied timing, ranging from 1 month to 3 years prior.

The main clinical characteristics of patients with MG and without MG are summarized in Table 1. Both groups were comparable in terms of baseline characteristics, but there was a higher prevalence of patients affected by previous neoplasms in the non-MG group (20% vs. 3.3%; *p* = 0.05).

Age categorization followed a ≥35-year cutoff, a threshold used in previous studies to differentiate early-onset vs. late-onset MG, given their potential clinical and immunological differences [3].

A total of thirty-nine (70.9%) procedures were carried out via a left-sided approach. For patients with MG, the mean operative time was 196.9 ± 79.9 min compared to 175.8 ± 61.6 min for non-MG patients (*p* = 0.285). Additionally, the MG group experienced a longer in-hospital stay (4.8 ± 2.6 vs. 3.3 ± 2.2 days, *p* = 0.01) and showed a significantly higher need for intensive care unit admission (*p* < 0.01) compared to the other group (Table 2).

No deaths were recorded in any of the groups. The incidence of conversion (3.3% vs. 4.0%, *p* = 0.895) and complications rates (*p* = 0.813) were comparable in the groups of patients with MG and non-MG thymomas. The number of extended resections (involving lung or pericardium) were similar in both groups (13.3% in patients with MG vs. 8.0% in non-MG patients; *p* = 0.850). No intraoperative haemorrhagic events or similar complications were observed, including on the right side of the dissection, despite theoretical concerns about vascular risks in this area. No cases of phrenic nerve palsy—neither right nor left—were recorded postoperatively.

A significantly higher proportion of patients with MG required postoperative radiotherapy (RT) compared to non-MG patients (60.0% vs. 32.0%, *p* = 0.03; Table 2).

In the multivariable analysis, the prognostic factors for conversion were vascular involvement (OR: 52.00, 95% CI: 1.726–1566.915, *p* = 0.023) and lung involvement (OR:15.45, 95% CI: 0.632–141.320, *p* = 0.040), as shown in Table 3.

The primary factor associated with complications was extended resections (OR:15.33, 95% CI:1.566–150.141, *p* = 0.019), while vascular involvement showed only a trend towards significance in the multivariable analysis (*p* = 0.075; Table 4). The only recorded complications were postoperative, with five patients (9.1%) developing atrial fibrillation and two patients (3.6%) experiencing a myasthenic crisis. Both complications were successfully managed, with no long-term sequelae.

No routine postoperative IVIG or plasmapheresis was required in our cohort.

**Figure 1:**
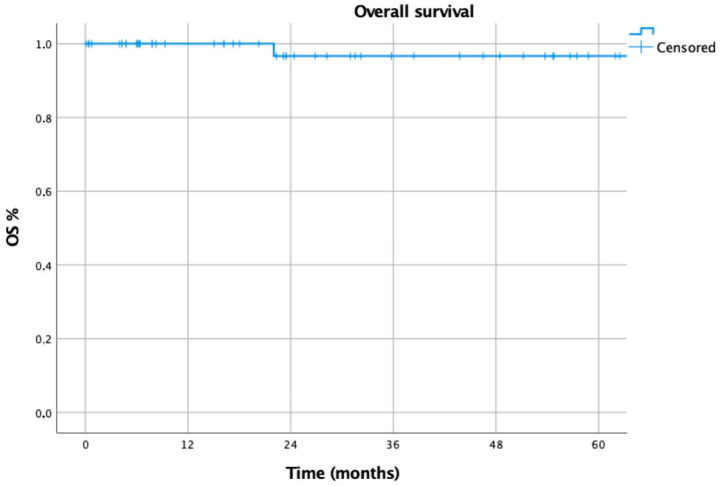
Overall survival of patients affected by thymoma.

**Figure 2:**
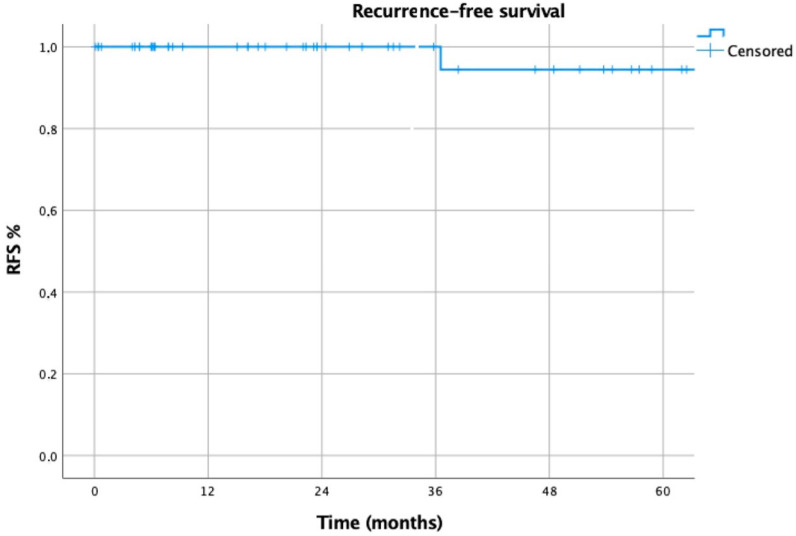
Recurrence-free survival of patients affected by thymoma.

The 2- and 5-year overall survival (OS) of the 55 patients affected by thymoma was 97% in both cases (Figure 1), while the 2- and 5-year recurrence-free survival (RFS) was 100% and 94%, respectively (Figure 2).

In the subgroup of MG thymomatous patients, the mean age at the time of surgery was 54.5 ± 15.9 years. After a mean follow-up period of 35.6 ± 25.7 months, 18 (60%) patients with myasthenia had an improvement in their clinical conditions. Among these, complete stable remission (CSR) was observed in 2 (6.6%) patients, pharmacological remission (PR) in 2 (6.6%), minimal manifestation (MM) in 12 (40.0%), and PR + MM in 4 (13.3%). Twelve patients (40%) exhibited no changes, maintaining a stable clinical condition. In particular, in patients with MG receiving preoperative corticosteroid therapy, nine patients (30.0%) had their dose reduced by half, and eight (26.7%) were kept on the same dose. Notably, eight patients (26.7%) were able to discontinue corticosteroids entirely following thymectomy.

Postoperative AChR antibody titers were not systematically assessed.

**Figure 3:**
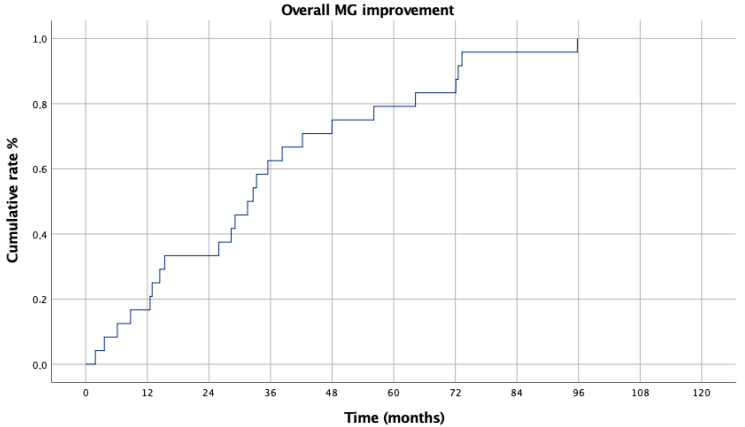
Overall MG improvement.

The overall improvement rate reached 38% at 2 years and increased to 83% at 5 years (Figure 3).

## 4. Discussion

This study examines the impact of robotic surgery in managing patients with thymoma, with or without MG, with an emphasis on postoperative surgical outcomes and long-term neurological results.

The European Society for Medical Oncology (ESMO) guidelines [27] (published in 2015) and the National Comprehensive Cancer Network (NCCN) guidelines consider minimally invasive surgery (MIS) a valuable option for thymic tumours, emphasizing the importance of achieving complete resection [28]. However, the currently available guidelines recommend MIS primarily for early-stage thymic tumours as there is limited published evidence supporting its use in more advanced stages.

Advancements in the robotic surgical technique have broadened the indications for robotic thymectomy. This approach enables the complete resection of advanced thymic epithelial tumours, including cases necessitating the concomitant resection of adjacent structures. Early clinical outcomes following robotic thymectomy for advanced thymic epithelial tumours have been promising [29]. Nevertheless, while the use of the robotic approach both in early and more advanced thymomas is expanding, studies investigating postoperative neurological outcomes in patients with thymoma-associated MG are still extremely limited [21,30,31,32].

In our series, R0 resection was achieved in eight patients with pathological stage III disease and in one patient with pathological stage IV disease according to the Masaoka–Koga staging system.

Similar data were reported by Romano et al. [21], who suggested that the tumour size should not be considered a contraindication for the robotic approach. They have, in fact, demonstrated the feasibility of treating Masaoka stage III thymomas and lesions with large diameters (up to 10 cm) (as in current experience) without complications. This was attributed to the precision afforded by robotic instruments, including their wide range of movement, the magnified three-dimensional view, and the Firefly mode (intraoperative use of fluorescence), which enhances the identification of critical structures such as the contralateral phrenic nerve. These features provide the possibility to perform a complete resection of the neoplasm, thymus, and perithymic fat, which are essential for the optimal treatment of thymoma and MG.

Similar results were reported by Huang [33] in 2024 in a study comprising 204 patients, where 55.9% had small thymomas and 44.1% had large thymomas. Although operative times were longer in the large thymoma group (*p* = 0.009), there were no significant differences in surgical parameters and postoperative outcomes between the two groups. In total, 190 patients (93.1%) successfully underwent complete tumour reception, classified as R0. The rates of R1 resection were 4.4% (ST) and 5.6% (LT), respectively, with no recorded cases of R2 resection. Additionally, four patients had an indeterminate margin status (Rx). There was no statistically significant difference in the rate of complete R0 resections between the two groups (94.7% vs. 91.1%, *p* = 0.41). Over a median follow-up period of 61.0 months (95% CI, 48.96–73.04), recurrence was identified in four patients (1.96%). Moreover, the group exhibited no significant differences in the 5-year overall survival (*p* = 0.25) or recurrence-free survival (*p* = 0.43).

In our experience, the majority of procedures were conducted using a left-sided surgical approach. The choice of access is influenced by the surgeon’s expertise as the extent of tumour involvement on one side. We prefer the left-sided approach as it provides superior visualization of the aortopulmonary window, a region where thymic tissue may reside. However, some surgeons opt for the right-sided approach, citing its advantage in clearly identifying the junction of the superior vena cava and innominate vein (9). In our practice, this junction is adequately visualized using the left-sided approach, enabling a more thorough removal of all thymic tissue and potentially improving the MG outcomes. The absence of intra- and postoperative complications, particularly phrenic nerve palsy and haemorrhagic events, in our cohort is noteworthy given that the left-sided RATS approach theoretically exposes the right phrenic nerve to higher risk due to its proximity to the superior vena cava and innominate vein. And the only two postoperative complications observed in this study—atrial fibrillation (9.1%) and myasthenic crisis (3.6%)—are known potential risks following thymectomy in patients with MG. While our findings reinforce the safety and feasibility of RATS, further studies in larger cohorts are needed to determine whether specific surgical modifications might further optimize phrenic nerve preservation and reduce conversion rates.

### 4.1. Postoperative Outcomes

In our experience, the mean operative time was 196.9 ± 79.9 min for patients with MG compared to 175.8 ± 61.6 min for those without MG (*p* = 0.285). Although this difference was not statistically significant, patients with MG had a significantly extended hospital stay (4.8 ± 2.6 vs. 3.3 ± 2.2 days; *p* = 0.01) and a markedly higher need for intensive care unit admission (*p* < 0.01) compared to the non-MG group. The longer operative time in patients with MG may be attributed to the “more precise” resection that we usually perform in this group of patients, including a meticulous skeletonization of the phrenic nerve bilaterally, often utilizing the Firefly mode for enhanced visualization.

The prolonged in-hospital stay and increased ICU requirement for MG patients reflect our institution policy, where patients with MG are routinely monitored in the ICU for one night postoperatively to ensure optimal management. Similar postoperative outcomes have been noted in previous studies. In the cohort reported by Kumar et al. [34], the median operative time was 180 min, with a median chest tube removal time of 1 day and a median hospitalization duration of 7 days (range: 3–30 days) in patients with thymoma. In their series, three complications were reported in the patients with thymoma-associated MG: two patients had a myasthenic crisis requiring mechanical ventilation and prolonged ICU stay (7 and 30 days), and one had hoarseness of voice, which resolved spontaneously.

Due to the extension of the disease in our patients, we performed an extended resections (involving lung or pericardium) with a similar percentage in both groups (13.3% in MG patients vs. 8.0% in non-MG patients; *p* = 0.850). Despite these extended operations, the conversion rates (3.3% vs. 4.0%, *p* = 0.895) and complications rates (minor complications), which were around 10% (*p* = 0.813), were similar in both MG- and non-MG patients with thymoma. These findings are consistent with the reported outcomes in robotic thymectomy, which demonstrates low conversion rates (<5%), low morbidity (<10%), and short hospital stays (<4 days) [32,34,35,36,37,38]. Mortality was null in our series, as in other reported studies [33,39,40]

### 4.2. Neurologic Results

In our cohort of patients with thymoma-associated MG, after an average follow-up period of 35.6 ± 25.7 months, 60% experienced clinical improvement of their condition. Specifically, complete stable remission (CSR) was observed in 6.6% of patients, pharmacological remission (PR) in 6.6%, and minimal manifestation (MM) in 40.0%, while a combination of PR + MM was observed in 13.3%.

In a study published in 2015, Keijzers et al. [31] investigated the neurological outcomes in patients with MG who presented with various thymic pathologies (including hyperplasia, cysts, lipomas, and thymomas) and underwent robotic surgery. Using the MGFA classification to assess both preoperative and post-intervention statuses, they observed a 3-year CSR of 5.6% in patients with thymoma.

In 2017, Kumar et al. [32] carried out an evaluation of oncological and neurological outcomes in 71 patients with MG who underwent robotic thymectomy for various thymic conditions. Their findings demonstrate that robotic surgery is both safe and feasible for the treatment of these patients, with an overall CSR rate of 38% and a mean follow-up time of 17.5 months. Among the 21 patients specifically with thymoma, the CSR rate reached 19%. Despite the substantial number of patients in this study and the favourable outcomes reported, the long-term neurological outcomes in patients with MG exclusively affected by thymoma remain inconsistent across studies.

Our study is one of the first to specifically focus on long-term neurological outcomes in a uniform cohort of patients with thymoma-associated MG. It has, in fact, well demonstrated that the progression and management of myasthenia gravis differs significantly between patients with thymoma and those with benign thymic disease [41]. Similarly, in the study conducted by Romano et al. [21], 53 patients with thymoma underwent robotic thymectomy using a fully endoscopic approach, which included 34 patients diagnosed with MG.

In this series, symptom improvement, as assessed by the MGFA-PIS criteria, was obtained in 76.5% of patients, with a CSR rate of 14.7% after a follow up of 36 months. These results appear to align with those of other studies [31,32] and our series.

After a mean follow-up time of 65.7 ± 43.1 months, Marcuse [39] further documented that CSR was achieved in 8.5% of patients, while PR was observed in 39.4%. Notably, patients with and without thymoma showed no statistically significant differences in accomplishing CSR or PR.

Our long-term clinical outcomes align with the most recent publications adopting the MGFA guidelines [19,38] for robotic thymectomy. However, it must always be kept in mind that the high variability of long-term results reported in many studies can be attributed to several factors, such as differences in follow-up durations and heterogeneous patient characteristics (e.g., ocular versus generalized MG, varying severity classes, differing preoperative symptom durations, racial diversity, and variable therapeutic schemes). Moreover, clinical evaluations in many studies were often conducted by multiple neurology teams, leading to a lack of standardized practices in drug prescription and post-thymectomy management strategies. On the contrary, in our study, clinical evaluation was always performed by the same two neurologists, ensuring a more homogeneous evaluation of MG outcomes using the MGFA-PIS. Additionally, only AChR-positive patients were included, while other MG subgroups, such as MuSK-positive, anti-titin-positive, or seronegative MG, were not studied. These subgroups may have different clinical behaviours, responses to thymectomy, and neurological outcomes, which remain unexplored in our cohort.

A higher necessity for postoperative radiotherapy was observed in the MG group compared to non-MG patients (60.0% vs. 32.0%, *p* = 0.03). The utilization of postoperative RT in our cohort followed standard oncological guidelines and was primarily driven by the tumour stage, histological classification, and resection status. Specifically, RT was more frequently used in patients with Masaoka–Koga stages III-IV disease, WHO B3 histology, or incomplete (R1/R2) resections, consistent with the findings reported by Florit et al. [40]. Patients with MG may have been treated more aggressively due to concerns about disease progression of a potentially more aggressive tumour behaviour, recurrence risk, or MG-related factor affecting oncological outcomes. Although the Masaoka–Koga stage and WHO histology did not differ significantly between the groups, MG-associated thymomas might have been perceived as a higher risk, leading to a more frequent RT recommendation even after complete resection (R0). There is a need for further studies in order to uniform RT guidelines in such way to improve long-term oncological outcomes in MG thymomatous patients.

In our analysis of risk factors associated with the surgical resection of thymoma in patients with MG, a multivariable analysis identified lung involvement (52.00 [1.726–1566.915], *p* = 0.023) and vascular involvement (15.45 [0.632–141.320], *p* = 0.04) as the primary factors for conversion. The primary factor associated with postoperative complications was extended resections (15.33 [1.566–150.141], *p* = 0.019); vascular involvement showed only a trend towards significance (*p* = 0.075).

A recent publication by Huang [33] supported these findings, highlighting that combined additional resections serve as an independent predictor of postoperative complications (HR, 2.61, 95% CI, 1.15–5.92, *p* = 0.022). However, the study did not identify any independent criteria that could reliably predict a patient’s risk of developing severe postoperative complications.

### 4.3. Limitations and Strengths

One of the primary limitations of this study lies in its single-centre, retrospective design. To gain a more comprehensive understanding of oncological outcomes, a longer follow-up period would be essential, particularly due to the slow and indolent growth of thymomas. Furthermore, in our MG cohort, only AChR-positive patients were included, without exploring the behaviour of patients in other MG subgroups (MuSK-positive, anti-titin-positive, or seronegative MG). Moreover, a prospective, multicentric study would be more suitable for thoroughly assessing both oncological and neurological outcomes.

The greatest strengths of this paper are instead represented by the fact that all operations were performed by a single skilled surgeon and all neurological pre- and postoperative evaluations were performed by the same two neurologists. This consistency ensures homogeneity in surgical treatment and in the evaluation of MG-PIS, providing reliable and comparable results.

## 5. Conclusions

The postoperative results of RATS for extended thymectomy for thymoma demonstrate its technical feasibility and safety. This minimally invasive approach is associated with low complication rates, shorter hospitalization periods, and satisfactory results, even in patients with a more advanced stage of thymoma and in patients with MG.

The significant neurological improvement in patients with thymomatous MG confirms the long-term clinical effectiveness of the robotic approach.

However, it is important to acknowledge that the successful implementation of the RATS approach requires significant surgical proficiency and experience and should be performed in specialized, high-volume centres that have access to comprehensive, multidisciplinary teams capable of ensuring the highest standard of care to optimize patients’ outcomes.

## Figures and Tables

**Table 1:** Clinical characteristics.

		MG (#30)	Non-MG (#25)	*p*
Sex (male)		10 (33.3%)	12 (48.0%)	0.269
Age (years)		54.7 ± 15.6	66.3 ± 11.3	0.003
Age (≥35 years)		26 (86.7%)	25 (100%)	0.06
Pathological Masaoka	I	7 (23.3%)	4 (16%)	0.382
	II	17 (56.7%)	18 (72%)	
	III	5 (16.7%)	3 (12(%)	
	IV	1 (3.33%)	0	
Histology (WHO)	A	3 (10%)	3 (12%)	0.289
	AB	5 (16.7%)	5 (20%)	
	B1	2 (6.7%)	1 (4%)	
	B1–B2	1 (3.3%)	2 (8%)	
	B2	7 (23.3%)	9 (36%)	
	B2–B3	8 (26.7%)	3 (12%)	
	B3	4 (13.3%)	2 (8%)	
Cardiovascular diseases		1 (3.3%)	3 (12.0%)	0.218
Comorbidities		17 (56.6%)	19 (76.0%)	0.133
Hypertension		10 (33.3%)	16 (64%)	
Type II diabetes mellitus		3 (10%)	2 (8%)	
Thyroiditis		4 (13.3%)	1 (4%)	
Previous neoplasms		1 (3.3%)	5 (20.0%)	0.05
Breast cancer		0	1 (4%)	
Prostate cancer		0	2 (8%)	
Colorectal cancer		0	2 (8%)	
Lung cancer		1 (3.3%)	0	

**Table 2:** Postoperative surgical outcomes.

	MG (#30)	Non-MG (#25)	*p*
Left approach	24 (80.0%)	15 (60.0%)	0.104
Surgical duration (min)	196.9 ± 79.9	175.8 ± 61.6	0.285
Hospital stay (days)	4.8 ± 2.6	3.3 ± 2.2	**0.01**
ICU admission	22 (73.3%)	1 (4.0%)	**<0.01**
Mortality	0%	0%	1.00
Conversion	1 (3.3%)	1 (4.0%)	0.895
Complications	3 (10.0%)	3 (12.0%)	0.813
Extended resections	4 (13.3%)	2 (8.0%)	0.850
Postoperative RT	18 (60.0%)	8 (32.0%)	**0.03**

**Table 3:** Uni- and multivariable analyses on prognostic factors for conversion.

Variables	Univariable Analysis	Multivariable Analysis	
	*p*-Value	OR [95% CI]	*p*-Value
Side	0.507		
Lung involvement	<0.001	52.00 [1.726–1566.915]	0.023
Vascular involvement	0.003	15.45 [0.632–141.320]	0.040
Pleural involvement	0.005		
Fat involvement	0.222		
Pericardial involvement	0.096		
Extended resections	0.018		
Conversion	0.071		
MG	0.895		

**Table 4:** Uni- and multivariable analyses on prognostic factors for complications.

Variables	Univariable Analysis	Multivariable Analysis	
	*p*-Value	OR [95% CI]	*p*-Value
Sex	0.596		
Side	0.478		
Lung involvement	0.071		
Vascular involvement	0.071	15.33 [0.757–310.393]	0.075
Pleural involvement	0.200		
Fat involvement	0.191		
Pericardial involvement	0.096		
Extended resections	0.009	15.33 [1.566–150.141]	0.019
Conversion	0.071		
MG	0.813		
ICU	0.665		
Steroids	0.528		
Plasmapheresis	0.348		

## Data Availability

No new data were created or analyzed in this study.
